# Gender‐Disaggregated Analysis of Sweetpotato Cultivation and Consumption in the Volta Region of Ghana

**DOI:** 10.1002/pei3.70097

**Published:** 2025-11-05

**Authors:** Liticia Effah‐Manu, Genevieve F. Opoku, Emmanuel Letsyo, Edem W. Anku, Hannah Moot

**Affiliations:** ^1^ Department of Food Science and Technology Ho Technical University Ho Ghana

**Keywords:** consumption pattern, gender, processing, production, sweetpotato

## Abstract

Women and men play a significant role in the cultivation and utilization of food crops. This study highlights the influence of gender on the cultivation and consumption of sweetpotato. The study employed cross‐sectional purposive and simple random sampling techniques to select 300 respondents from three district capitals of the Volta region of Ghana. From the results, land preparation, planting, weeding and fertilizer application are dominated by men, while women are more involved in harvesting (34%) and marketing (74.3%). Gender preferences showed that women like the orange‐fleshed variety more than men. More women household heads (40%) and individuals (32.2%) frequently consume sweetpotatoes compared to men, 31.1% and 26.6%, respectively. The consumption was linked to affordability, accessibility and preference. The smell of the sweetpotato strongly influenced the consumption pattern. Flour was the most preferred food product by women, while chips (frozen) were liked by men. The form of eating sweetpotato was positively correlated with the level of education, marital status and age, with significance at *p* < 0.05. Generally, the cultivation of sweetpotatoes in the districts is declining. Production and consumption intervention strategies such as policy implementation and nutrition education programs are crucial for increasing the consumption of sweetpotato. This is particularly important for the orange‐fleshed variety, enhancing food security efforts.

## 1 Introduction

Sweetpotato ( *Ipomoea batatas* ) is a root crop that belongs to the Convolvulaceae family. It consists of many varieties and is native to tropical parts of America with purplish flowers and large nutritious roots (Swami 2024). In Ghana and parts of West Africa, it is referred to as a secondary crop because it complements the major root crops like cassava and yam (Kofi Annan Foundation 2019). This is because, sweetpotato is not a principal agricultural crop in any of the regions according to SRID (2022). The skin colors range from white, cream, yellow, orange, pink, red to purple and the flesh may be white, cream, yellow, orange or purple (Shekhar et al. 2015). Sweetpotato is a superfood (Islam 2024) and a food security crop (Motsa et al. 2015). It outranks most staples in pro‐Vitamin A, dietary fiber, protein content, other vitamins and minerals (Burri 2011; Motsa et al. 2015). With their short harvest time, sweetpotatoes have a profound benefit for household food and nutritional security combating vitamin A deficiency in many consuming communities (HarvestPlus 2012).

The Volta region has fertile agricultural lands producing maize, cassava, yam, oil palm, plantain and export crops like pineapple, mangoes and cocoa. Also, the region is among the major regions in Ghana, cultivating sweetpotatoes in addition to Eastern, Central, Greater Accra and Upper East (Bidzakin et al. 2014). It is considered as having the highest implied food insecure populations, ranging between 200,000 and 300,000 (Osei 2024). These statistics bring out the importance of understanding cultivation trends and consumption of the nutrient‐rich sweetpotato in the region. In Ghana, a micronutrient survey revealed 30% of pre‐school children suffer from iron and vitamin A deficiency, while 19% of children are stunted and underweight (11%) (GDHS 2014). In terms of anemia cases, the Volta region has the highest (Yawson et al. 2017). The authors attribute the findings to the less than satisfactory consumption of foods rich in proteins and vitamin A. Evidence has shown that the consumption of orange‐fleshed sweetpotato can significantly improve vitamin A intake among vulnerable populations (Truayinet 2020). In Mozambique, it accounted for 75% of total vitamin A intake among women and children who participated in a large‐scale intervention study (Backiny‐Yitna and McGee 2015). Also, a randomized controlled trial in South Africa showed that the consumption of 125 g of orange‐fleshed sweetpotato over a period of 53 school days improved liver vitamin A stores in 5 to 10‐year‐old children (Truayinet 2020). According to Okyere et al. (2022), there is a need for targeted interventions on vitamin A supplementation considering trends in Ghana. This has terrible consequences for human health and well‐being and has direct implications on economic productivity (Haddad 2013). Intake of food staples like sweetpotatoes and specifically the orange‐fleshed variety can complement efforts by the government in the fight against micronutrient deficiencies. Although sweetpotatoes are being grown in the country, data from FAOSTAT (2020) indicate marginal growth in production across the country. Existing literature after the year 2019 shows little or no information on empirical studies on gender‐based issues in cultivation, consumption patterns as well as processing preferences. This work is important to make available information on the hindrances to increasing cultivation and consumption of sweetpotatoes in Ghana, specifically, the Volta region.

The gender involved in cultivation and gender‐based preferences could affect cultivation and consumption patterns. Recent studies have revealed that gender plays a key role in the production and consumption of food crops (Truayinet 2020; Backiny‐Yitna and McGee 2015). Gender‐based roles and attributes change over time and vary with different cultural contexts. As a consequence, men and women are affected differently by factors such as marketing and socio‐cultural environments. Factors such as access to control over and utilization of resources have been major indicators of gender differences (Kassie et al. 2014; Wekwete 2013). Household heads have a significant influence on the adoption of potato varieties (Tanellari et al. 2014). Mugonolaa et al. (2013) for instance have noted that in fertilizer applications for improved yield, male‐headed households have a higher probability of participation than female‐headed households. Moreover, in the adoption of hybrid seeds, Namonje‐Kapembwa and Chapoto (2016) found that female farmers are less likely to adopt or use fertilizer on their farms compared to male counterparts. These gender‐related disparities can greatly affect food cultivation and consumption. Recent findings in the literature show disparities in the consumption of certain foods. Investigating gender preferences will help to tailor interventions to the needs of specific groups in the sweetpotato value chain. Taste differences and preferences among men and women will allow for personalized nutritional strategies that can improve nutritional deficiencies in their homes.

Significant differences in food intake by gender exist (Nasreddine et al. 2020; Feraco et al. 2024). These differences can have either a positive or negative effect on food choices and nutritional intake of many households of a particular demographic. For example, the differences in quantity and quality of food intake may contribute to under‐nutrition (Mkandawire et al. 2022). Moreover, households which are headed by either a man or a woman will influence the type of food consumed in those households, thus leading to nutritional disparities (Katapa 2006). Although there have been many attempts by stakeholders to combat malnutrition and vitamin A deficiency by promoting the consumption of bio‐fortified varieties of sweet potatoes in regions that cultivate them, there has not been much data to track the consumption pattern and factors that influence consumption. The study therefore aimed to fill these gaps by considering gender‐based differences in cultivation and consumption, and processing preferences. Understanding these differences is essential in prioritizing cultivation interventions as well as specific processing needs.

## 2 Methods

### 2.1 Study Site and Characteristics

**FIGURE 1:**
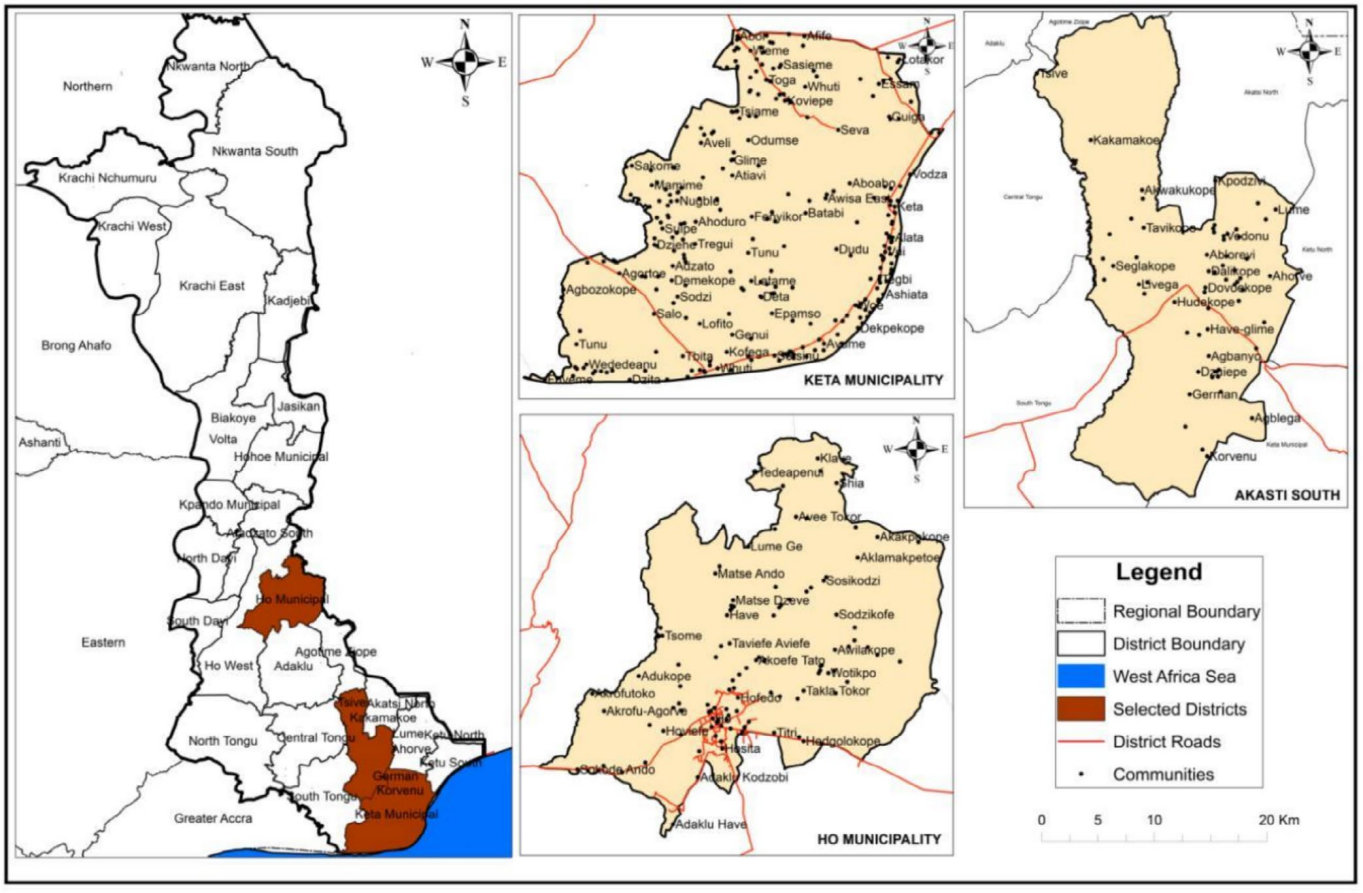
Map of study region and districts. Authors created the map.

The study was conducted in three district capitals of the Volta region of Ghana: Akatsi South, Ho and Keta (Figure 1). These districts are significant for the production of sweet potatoes in the Volta region. Akatsi, Ho and Keta are the capital cities for the three districts where the work was conducted. These cities have high population densities since they serve as economic hubs and service provision centres. They therefore link most rural communities where many consumers aggregate. In addition, these cities are characterized by varying cultural attitudes that can influence consumption and preferences for sweetpotatoes.

The Akatsi South municipality is located between latitudes 60° S and 70° N and longitudes 00° W 10 10° E, whereas the Keta municipal district lies within longitudes 0.30° E and 1.05° E and latitudes 5.45° N and 6.005° N. The Ho municipal district on the other hand has Ho as its capital which also serves as the economic hub of the Volta region. The Municipality is located between latitudes 6°20″ N and 6°55″ N and longitudes 00°12′ E and 00°53′ E. The Keta Municipal district lies within longitudes 0.30° E and 1.05° E and Latitudes 5.45° N and 6.005° N (Ghana Statistical Service 2010).

### 2.2 Sampling and Sample

A combination of cross‐sectional purposive and simple random sampling was employed in the selection of respondents. One hundred (100) respondents from each district capital were used, making a total of 300. Data was gathered from sweetpotato consumers, made up of farmers and traders using a semi‐structured questionnaire. Questionnaire administration was done on market days when most farmers had converged in the markets. While most of the respondents were able to fill in the questionnaires on their own, others were assisted to do the same. Close supervision was provided during the data collection process to address all technical problems in the field.

### 2.3 Ethical Considerations and Approval

The respondents volunteered to participate in this study after verbal informed consent was sought prior to the commencement of data collection. Ethical approval was received from the Ethical Review Committee of Ho Technical University (HTU/DRI/EC2022‐01).

### 2.4 Data Analysis

The study employed the use of descriptive statistics (frequency with percent), contingency tables (cross tabulation) with the chi‐square test and a binary logistics regression model to analyze the data. The Statistical Package for Social Science (SPSS) version 23 was used in the analysis. The results were then presented in tables and charts, from which inferences were drawn.

## 3 Results

### 3.1 Socio‐Demographic Information

The respondents were 51.3% men and 48.7% women, with their main educational levels being secondary (37.3%) and tertiary (33.7%). About 18.3% had basic education while the remaining 10.7% had no formal education. The majority of the respondents (57.3%) were single while 33.5% were married. The age range showed 45.0% between 18–30 years, 31–40 years (20.5%), and 41–50 years (13.4%). The extreme age groups below 18 years and above 51 years were 14.4% and 6.7%, respectively (Table 1).

**TABLE 1:** Socio‐demographic information of sweetpotato actors from three districts of the Volta region.

District	Frequency	Farmer (%)	Trader (%)
Akatsi	100	43 (43)	57 (57)
Ho	100	42 (42)	58 (58)
Keta	100	42 (42)	58 (58)
Education			
No formal education	32	18 (56.25)	14 (43.75)
Basic education	55	10 (18.20)	45 (81.8)
Secondary education	112	70 (62.5)	42 (37.5)
Tertiary education	101	36 (35.64)	65 (64.36)
Marital status			
Single	161	76 (47.20)	85 (52.80)
Married	112	82 (73.21)	30 (26.79)
Widowed	22	12 (54.55)	10 (45.45)
Divorced	5	5 (100)	0
Age			
Below 18 years	43	20 (46.5)	23 (53.5)
18–30 years	134	69 (51.5)	67 (48.51)
31–40 years	63	35 (55.56)	28 (44.44)
41–50 years	40	18 (45)	22 (55)
51 years and above	20	4 (20)	16 (80)

### 3.2 Sweetpotato Cultivation

Involvement of both females and males in the cultivation of sweetpotatoes was analyzed among the farmers who participated in the research. Out of the 300 respondents, 39% (117) were farmers and 61% (183) traders, and all of them were consumers of sweetpotatoes. Land preparation, planting, weeding, fertilizing and harvesting were undertaken mainly by males, while the females were mainly involved in the marketing of the sweetpotatoes. Land preparation is a vital element and a prerequisite for sweetpotato production. A high percentage of men (92%) were involved in land preparation compared to 17% reported for women. This clearly indicates that land preparation is heavily dominated by them. Planting as well as weed control and fertilizer application were also dominated by men (80% and 81% respectively). Nevertheless, harvesting showed a slight increase in women's participation (34%).

#### 3.2.1 Challenges Faced by Respondents

**FIGURE 2:**
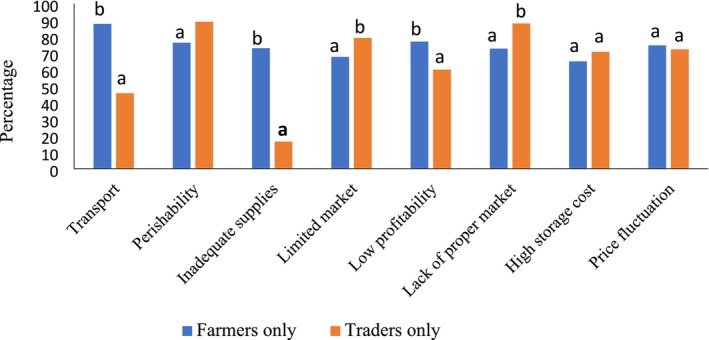
Challenges faced by sweetpotato farmers and traders. Values under same title with different letters are significantly different (*p* < 0.05).

Figure 2 shows the challenges encountered by the respondents (i.e., farmers and traders) in the cultivation and marketing of sweetpotatoes. Generally, transportation issues, inadequate supplies, price fluctuation and high storage costs are among the dominant factors affecting the cultivation and marketing of sweetpotatoes in the studied districts. Major challenges peculiar to farmers include transportation, perishability, price fluctuation and associated low profitability, whilst for traders, their major challenges include lack of a proper market, limited market, perishability and price fluctuation. Significant differences existed between the perceptions of farmers only and traders only for all the challenges except high storage costs and price fluctuations.

#### 3.2.2 Demand for Sweetpotato in the Past 3 Years and Consumption Patterns

**FIGURE 3:**
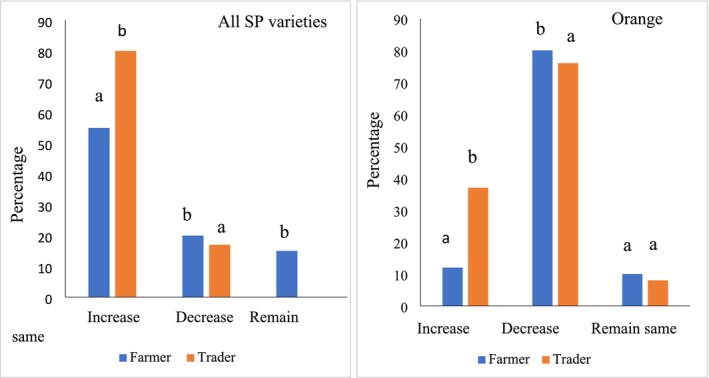
Demand for all varieties of sweetpotatoes and only orange‐fleshed sweetpotatoes in the past 3 years. Values under same title with different letters are significantly different (*p* < 0.05).

The demand for sweetpotatoes in general, and orange‐fleshed sweetpotatoes specifically by farmers and traders in the past 3 years was examined (Figure 3). Generally, more traders compared to farmers (80% vs. 55%) are of the view that the demand for sweetpotatoes in the past 3 years has been increasing. However, demand for orange‐fleshed sweetpotatoes has decreased in the past 3 years as seen in Figure 3.

Further, the study identified the frequency at which respondents consumed sweetpotato individually and for households, as well as knowledge of the nutrient content of sweetpotato (Table 3). The study observed that 43.7% of the respondents were household heads made up of men (36.6%) and women (7.1%) who were responsible for making the decisions at home and making money available. In terms of consumption patterns, 35.7% of individuals consume sweetpotato once a week, three times a week (22.9%) and every day (22.9%), when it is in season.

**FIGURE 4:**
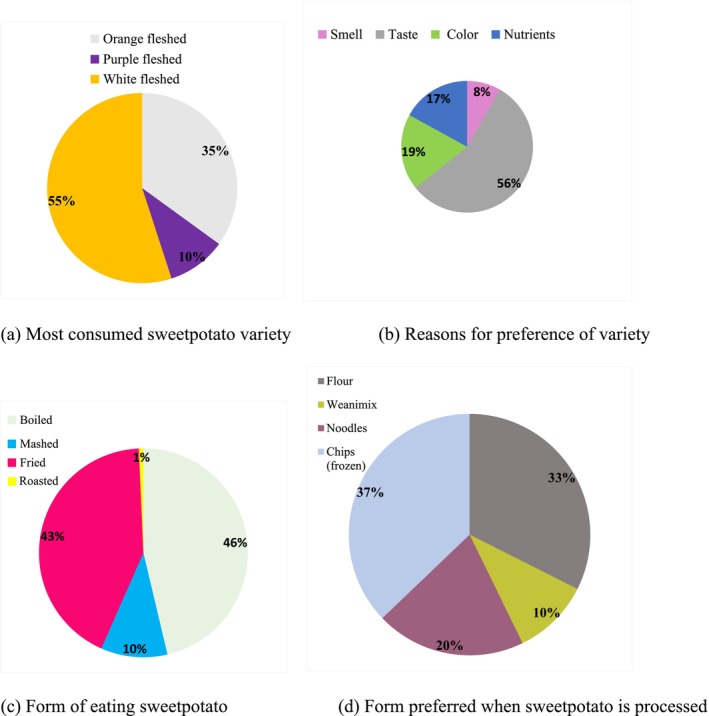
Most consumed variety with reasons (a, b) and the preferred forms of eating unprocessed and processed sweetpotatoes (c, d).

For consumption at the household level, 30.6% consumed sweetpotato once a week, 23.0% consumed sweetpotato three times a week, while 35.4% consumed sweetpotato every day (especially when it is in season). The reason for consuming sweetpotato was mainly as a result of affordability, accessibility and likeness. The majority of the respondents (81.3%) know that orange‐fleshed sweetpotato is high in nutrients (Table 2). When asked about the nutrients, they indicated carbohydrates, vitamins A, B, C and B_6_, protein, potassium, and minerals as the major nutrients in orange‐fleshed sweetpotatoes (Figure 4).

**TABLE 2:** Sweetpotato consumption patterns in the producing districts.

	Frequency	Percent (%)
Head of home		
Yes	125	43.7
No	161	56.3
Total	286	100
How often you consume sweetpotato		
Not at all	36	12.1
Once a week	106	35.7
Three times in a week	68	22.9
Every day (when it is in season)	90	29.3
Total	300	100.0
How often household consume sweetpotato		
Not at all	32	11.0
Once a week	98	30.6
Three times in a week	67	23.0
Every day (when it is in season)	103	35.4
Total	300	100.0
Reasons		
Accessible	100	33.3
Cheap	116	38.7
Liked by all	84	28.0
Expensive	300	100.0
Knowledge of high nutrients in orange‐fleshed sweetpotatoes		
Yes	235	81.3
No	54	18.7
Total	289	100.0

From the data, the majority of the participants consumed white‐fleshed sweetpotato representing 55.0%, followed by orange‐fleshed (35.0%) and purple (10%). The reason for consuming a particular variety of sweetpotato was mainly due to its taste, as indicated by 56.0% of the respondents. Boiling (46.3%) and frying (42.7%) are the main forms respondents prefer their sweetpotatoes. Gender‐based preferences showed that more females like the orange‐fleshed sweetpotatoes while more males like the white‐fleshed. The taste is most important to all respondents in the selection of sweetpotato variety. In terms of processing, respondents prefer chips (frozen) (32.3%) and flour (37%). Gender‐wise, flour from sweetpotato is the most preferred by women, whilst chips (frozen) are liked by men. These preferences are linked to physiological differences in men and women, access to information on sweetpotatoes and economic power (Table 5).

From Table 3, color had no significant effect on the reason for consuming sweetpotato. Age had a significant influence on the reason for consuming sweetpotato by the respondents. Respondents within the age bracket of 51 years or more were 10 times more likely to consume sweetpotato due to its nutrients than respondents within the age bracket < 18 years. There is therefore a need to educate young people on the nutrients in sweetpotatoes. Farmers were three times more likely to consume sweetpotato because of its nutrients than those who were not farmers, OR = 3.582, *p*‐value < 0.05.

**TABLE 3:** Logistic regression model for demographic characteristics and reason for consuming sweetpotato variety.

	Aroma		Taste		Color		Nutrient	
	Estimate	OR	Estimate	OR	Estimate	OR	Estimate	OR
Gender: Female	−0.37	0.691	−0.443	0.642	0.378	1.460	0.158	1.171
Educational level								
Basic education	−0.102	0.903	−0.046	0.955	−0.056	0.945	−0.025	0.976
Secondary education	0.538	1.713	−0.485	0.616	−0.404	0.668	−0.265	0.767
Tertiary education	0.938	2.556	−0.967	0.380	−0.071	0.931	0.294	1.342
Marital status								
Married	1.392	4.024**	0.733	2.082	−0.498	0.608	−0.617	0.539
Widowed	2.654	14.215*	−0.021	0.980	0.240	1.271	−1.228	0.293
Divorced	−14.151	0.000	−0.402	0.669	0.339	1.403	−0.231	0.794
Age								
18–30 years	−0.512	0.600	0.655	1.926	−0.517	0.596	0.384	1.469
31–40 years	−1.328	0.265	0.244	1.276	−0.283	0.753	0.984	2.675
41–50 years	−2.143	0.117	0.218	1.244	0.697	2.008	0.368	1.444
51 years and above	−20.957	0.000	−0.777	0.460	−0.478	0.620	2.364	10.634**
Prediction accuracy	92.50%		65.20%		79.50%		79.20%	
Omnibus tests of model coefficients	—							
	17.136		38.765***		14.385		21.104	
Hosmer and Lemeshow test	—							
	5.717		11.260		11.431		10.993	
Cox & Snell *R* ^2^	0.060		0.130		0.050		0.073	
Nagelkerke *R* ^2^	0.140		0.175		0.079		0.112	

Abbreviation: OR, odds ratio.

* OR significant at 0.10 (10%).

** OR significant at 0.05 (5%).

*** OR significant at 0.01 (1%).

Concerning the smell of the sweetpotato, married respondents were four times more likely to consume sweetpotato because of its smell, OR = 5.024, *p*‐value < 0.05 than singles. It was also observed that those who were consumers were seven times more likely to eat sweetpotato because of smell, OR = 7.127 and *p*‐value < 0.05. Marketers were also four times more likely to consume sweetpotato because of the smell than those who were not marketers. The respondents who were farmers were less likely to consume sweetpotato because of its taste, OR = 0.070, *p*‐value < 0.05.

### 3.3 Demographic Characteristics and Consumption Pattern

The cross tabulation and chi‐square test with Cramer's V were used to assess the distribution, level of association and effect size. There was a significant association between the gender of respondents and household consumption patterns (Table 4). With a *p*‐value < 0.05 and a high effect size of 0.181, there was a high association between gender and the consumption patterns of households. This is due to the fact that gender is a key influence on household food consumption and nutritional intake. From the table, females consume sweetpotato more frequently than males, suggesting that females prefer sweetpotatoes to their male counterparts.

**TABLE 4:** Consumption pattern of respondents.

Dependent	Factors	Consumption pattern				Chi‐square value	N‐N
		Not at all	Once a week	Three times in a week	Every day (when it is in season)		Cramer's *V*
	Gender						
Individual consumption	Man	16 (10.4)	54 (35.1)	43 (27.9)	41 (26.6)	5.134	0.131
	Woman	20 (14.0)	52 (36.4)	25 (17.5)	46 (32.2)		
Household consumption	Man	12 (7.9)	48 (31.8)	44 (29.1)	47 (31.1)	9.517**	0.181
	Woman	20 (14.3)	41 (29.3)	23 (16.4)	56 (40.0)		
	Education level						
Individual consumption	No formal education	4 (12.5)	5 (15.6)	7 (21.9)	16 (50.0)	27.210***	0.175
	Basic education	3 (5.6)	18 (33.3)	16 (29.6)	1 (31.5)		
	Secondary education	10 (8.9)	37 (33.0)	31 (27.7)	34 (30.4)		
	Tertiary education	19 (19.2)	46 (46.5)	14 (14.1)	20 (20.2)		
Household consumption	No formal education	3 (9.4)	5 (15.6)	89 (25.0)	16 (50.0)	36.741***	0.205
	Basic education	2 (3.7)	14 (25.9)	17 (31.5)	21 (38.9)		
	Secondary education	5 (4.6)	32 (29.4)	29 (26.6)	43 (39.4)		
	Tertiary education	22 (22.9)	38 (39.6)	13 (13.5)	23 (24.0)		
	Marital status						
Individual consumption	Single	27 (17.1)	62 (39.2)	36 (22.8)	33 (20.9)	27.769***	0.182
	Married	5 (5.3)	33 (35.10)	21 (22.3)	35 (37.2)		
	Widowed	4 (18.20)	3 (13.6)	5 (22.7)	10 (45.5)		
	Divorced	—	—	—	4 (100.0)		
Household consumption	Single	24 (15.5)	50 (32.3)	33 (21.3)	48 (31.00)	18.058**	0.149
	Married	5 (5.5)	28 (30.8)	25 (27.5)	33 (36.3)		
	Widowed	2 (9.1)	4 (18.2)	4 (18.2)	12 (54.5)		
	Divorced	—	—	—	4 (100.0)		
	Age						
Individual consumption	Below 18 years	6 (14.3)	14 (33.3)	12 (28.6)	10 (23.8)	34.439***	0.197
	18–30 years	21 (15.9)	56 (42.4)	32 (24.2)	23 (17.4)		
	31–40 years	3 (4.9)	23 (37.7)	14 (23.0)	21 (34.4)		
	41–50 years	5 (12.5)	11 (27.5)	7 (17.5)	17 (42.5)		
	51 years and above	1 (5.0)	2 (10.0)	3 (15.0)	14 (70.0)		
Household consumption	Below 18 years	3 (7.1)	13 (31.0)	15 (35.7)	11 (26.2)	27.935***	0.179
	18–30 years	22 (17.2)	42 (32.8)	26 (20.3)	38 (29.7)		
	31–40 years	4 (6.8)	18 (30.5)	18 (30.5)	19 (32.2)		
	41–50 years	2 (5.0)	13 (32.5)	6 (15.0)	19 (47.5)		
	51 years and above	1 (5.0)	3 (15.0)	2 (10.0)	14 (70.0)		

* Chi‐square of association significant at 0.10 (10%).

** Chi‐square of association significant at 0.05 (5%).

*** Chi‐square of association significant at 0.01 (1%).

For individuals and at the household level, respondents' educational level had a significant association with sweetpotato consumption pattern; associated chi‐square *p*‐value < 0.05. Respondents with no formal education consume sweetpotato more frequently than those with formal education. The divorced and widowed also consume sweetpotatoes more frequently than married and single respondents. There was a significant association between the age of participants and the consumption pattern. It was observed that the older age bracket consumed more sweetpotatoes than the younger age group at the individual and household level.

The association between gender and other background characteristics of participants and the form in which the sweetpotato was consumed was examined and the results are presented in Table 5. A significant association existed between participants' educational level and the form of consuming sweetpotato, chi‐square value of 19.403 and *p*‐value < 0.05. The effect size was medium (Cramer's *V* = 0.148). This suggests that the form of eating sweetpotato was correlated with the level of education. Thus, respondents with a low level of education (no formal education to secondary education) preferred boiled sweetpotato while participants with a higher level of education (tertiary) preferred fried sweetpotato.

**TABLE 5:** Sociocultural characteristics and form of consuming sweetpotatoes.

	Form of consuming sweetpotatoes					Cramer's *V*
	Boiled	Mashed	Fried	Other		
Gender						
Male	80 (53.0)	10 (6.6)	60 (39.7)	1 (0.7)	6.896*	0.153
Female	59 (41.3)	21 (14.7)	62 (43.4)	1 (0.6)		
Education level						
No formal education	21 (67.7)	5 (16.1)	5 (16.1)	—	19.403**	0.148
Basic education	27 (50.9)	6 (11.3)	20 (37.7)	—		
Secondary education	55 (50.0)	13 (11.8)	41 (37.3)	1 (0.9)		
Tertiary education	36 (36.0)	7 (7.0)	56 (56.0)	1 (1.0)		
Marital status						
Single	58 (37.2)	13 (8.3)	85 (54.5)	—	53.616***	0.255
Married	59 (62.8)	14 (14.9)	21 (22.3)	—		
Widowed	10 (47.6)	4 (19.0)	5 (23.8)	2 (9.5)		
Divorced	3 (75.0)	—	1 (25.0)	—		
Age						
Below 18 years	12 (30.0)	3 (7.5)	25 (62.5)	—	32.977***	0.194
18–30 years	56 (42.4)	10 (7.6)	66 (50.0)	—		
31–40 years	36 (59.0)	10 (16.4)	15 (24.6)	—		
41–50 years	23 (57.5)	5 (12.5)	11 (27.5)	1 (2.5)		
51 years and above	10 (52.6)	3 (15.8)	5 (26.3)	1 (5.3)		

* Chi‐square of association significant at 0.10 (10%).

** Chi‐square of association significant at 0.05 (5%).

*** Chi‐square of association significant at 0.01 (1%).

There was also a significant association between respondents' marital status and the form of eating sweetpotatoes. This is explained by a chi‐square value of 53.616, a *p*‐value of < 0.01, and a large effect size (≥ 0.25). This means that marital status had a very high effect on the form of eating sweetpotato, as the singles preferred fried sweetpotatoes while the married, divorced and widowed preferred the boiled sweetpotato. A significant association was also found between the age groups of respondents, the form of sweetpotatoes they consumed (*p*‐value < 0.01) and had a medium to large effect size. Ages below 18 years and 18–30 years often consume fried sweetpotatoes while those above 30 years prefer boiled sweetpotatoes. Therefore, the chi‐square values with associated *p*‐values < 0.05 showed a significant association between respondents' educational level, marital status, and age group and the form of sweetpotatoes they consumed.

#### 3.3.1 Socio‐Demographic Characteristics and Preferred Form of Orange‐Fleshed Sweetpotato When Processed

In Table 6, results of the association between background characteristics of the respondents and the form of sweetpotatoes preferred when processed are presented. It was observed that 35.8% of the participants preferred chips (frozen), flour (33.1%), noodles (20.5%) and weaning mix (weanimix, 10.6%). Cross tabulation with the chi‐square test and Cramer's *V* effect size was used to examine the association and the level of effect of background characteristics on the preference of the form of processed sweetpotatoes. It was observed that the educational level of participants, marital status and age had a significant effect on the preference for sweetpotatoes when processed.

**TABLE 6:** Socio‐demographic characteristics and preferred form of processed sweetpotatoes.

	Form preferred to have the sweep potatoes when it is processed					Cramer's *V*
	Flour	Weanimix	Noodles	Chips (frozen)		
Gender						
Male	54 (35.8)	1 (11.3)	30 (19.9)	50 (33.1)	1.501	0.072
Female	43 (30.3)	14 (9.9)	30 (21.1)	55 (38.7)		
Education level						
No formal education	18 (58.1)	1 (3.2)	5 (16.1)	7 (22.8)	28.168***	0.179
Basic education	14 (26.4)	9 (17.0)	9 (17.0)	21 (39.6)		
Secondary education	47 (42.7)	11 (10.0)	20 (18.2)	32 (29.1)		
Tertiary education	18 (18.2)	10 (10.1)	26 (26.3)	45 (45.5)		
Marital status						
Single	34 (21.8)	20 (12.8)	30 (18.2)	72 (46.2)	35.698***	0.208
Married	47 (50.5)	5 (5.4)	20 (21.5)	21 (22.6)		
Widowed	9 (42.9)	1 (4.8)	5 (23.8)	6 (28.6)		
Divorced	1 (25.0)	2 (50.0)	—	1 (25.0)		
Age						
Below 18 years	7 (17.5)	10 (25.0)	6 (15.0)	17 (42.5)	29.180***	0.183
18–30 years	36 (27.3)	13 (9.8)	27 (20.5)	56 (42.4)		
31–40 years	27 (44.3)	4 (6.6)	15 (24.6)	15 (24.6)		
41–50 years	21 (52.5)	2 (5.0)	7 (17.5)	10 (25.0)		
51 years and above	4 (22.2)	2 (11.1)	5 (27.8)	7 (38.9)		

* Chi‐square of association significant at 0.10 (10%).

** Chi‐square of association significant at 0.05 (5%).

*** Chi‐square of association significant at 0.01 (1%).

There was a significant association between educational level and the form of sweetpotatoes preferred when processed; the chi‐square value was 28.168, *p*‐value < 0.05. The size of the effect according to Cramer's *V* was medium; Cramer's *V* = 0.179. This suggested that participants with no formal and secondary education preferred sweetpotatoes in the form of flour, while participants with tertiary education preferred chips (frozen). Also, married and widowed participants preferred flour while singles preferred chips showing a significant association and medium to large effect size (*p*‐value < 0.05).

The age groups of the respondents showed a significant association with the form of sweetpotatoes preferred when processed. From the results, a chi‐square value of 29.180 and a *p*‐value of < 0.05 showed a significant association. The effect size was medium; Cramer's V of 0.183. From the distribution, ages between 31–40 and 41–50 years preferred flour while ages below 31 years preferred chips (frozen). It was observed generally that there was an association between respondents' educational level, marital status and age group and the form of sweetpotatoes preferred when processed. Higher levels of education participants preferred chips (frozen) while lower levels of education preferred flour. Furthermore, the singles preferred chips while married and widowed preferred flour, whereas respondents below the age of 31 years preferred chips.

## 4 Discussion

The results suggest that men and women dominate the cultivation and marketing of sweetpotato, respectively. Planting as well as weed control and fertilizer application were also dominated by males. One male individual expressed the view that “the women might waste some of the fertilizer” since they lack expertise in fertilizer application. There were other similar comments. This is also due to the fact that most of the farm land is owned and managed by men in these areas. In Northern Nigeria, David (2015) found that though sweetpotato is a source of income for women, gender‐related structural constraints restrict them from engaging in large‐scale production. In relation to harvesting, some men interviewed hold the belief that “women are more meticulous in harvesting, hence, they hardly damage the sweetpotato during harvesting”. Marketing, as the Ghanaian traditional system portrays it, has always been the preserve of women. It was therefore not surprising that women dominated (74.3%) the marketing side of production akin to a study in Northern Uganda, where sweetpotato marketing was dominated by women (Mudege et al. 2016). In Uganda, work done by Mdege et al. (2022) found that while Senyange women were in charge of all farming activities and could decide where to sell, Bugulumbya men, on the other hand, were fully in charge of marketing. Although Amengor et al. (2016) demonstrated dominance for men in land preparation and planting, they found that females dominated in fertilizer application, harvesting and marketing. Male dominance in production was also realized by Sanoussi et al. (2016). In Nigeria, Gbigbi (2019) identified a contrasting view that production and marketing of sweetpotatoes were dominated by females. Gendered norms cause people to subscribe to the general notion that commercial activities, are the preserve of men, while women's food‐related activities are dedicated to feeding their families (Rao 2020). Rao (2020) additionally found that lack of support in terms of planting materials, markets and sale of products are some limiting factors for female sweetpotato farmers.

In terms of the challenges, similar observations were made in a study by Zawedde et al. (2014) in Kapondo, Kenya. He found a list of challenges including high cost of transport, high perishability, low demand, low pricing and lack of storage facilities among others. In this present study, the farmers reported higher “low profitability” (77%) compared to traders (60%). A similar observation was made by Amengor et al. (2016) where traders made more profit than farmers. In the recommendation by Amengor et al. (2016), sweetpotato farmers in these districts could form cooperatives to have better bargaining power for prices and inputs.

As indicated by the respondents, Sanoussi et al. (2016) also found that calcium, magnesium, potassium, phosphorus, iron, zinc and sodium are the dominant minerals present in orange‐fleshed sweetpotatoes. According to Girard et al. (2017) and Kurabachew (2015), the orange‐fleshed sweetpotatoes can significantly contribute to a reduction in vitamin A deficiency. Furthermore, different vitamins, antioxidants and non‐digestible dietary fiber are present in the orange‐fleshed sweetpotatoes (Endrias et al. 2016; Rodrigues et al. 2016). The taste as a determining factor in the choice of sweetpotato variety correlates positively with a study by Zawedde et al. (2014) who found that good taste is an important characteristic for maintaining the growth of sweetpotato varieties. Though sweetpotatoes have varying smells (Leksrisompong et al. 2011), smell was the least (8.3%) among the reasons for consuming sweetpotatoes.

Respondents within the age bracket of 51 years or more were 10 times more likely to consume sweetpotato due to its nutrients than respondents within the age bracket of < 18 years. This could be attributed to knowledge gained by the elderly concerning sweetpotatoes compared to the younger folks. Mmasa and Msuya (2012) also found that 34% of sampled respondents consume sweetpotato because of its nutrients. There is therefore a need to educate young people on the nutrients in sweetpotatoes. Farmers were three times more likely to consume sweetpotato because of its nutrients than those who were not farmers, OR = 3.582, *p*‐value < 0.05. This finding suggests that sweetpotato farmers in these districts have the requisite knowledge of the crop they cultivate.

Females consuming more sweetpotatoes could be attributed to the low cost and availability of sweetpotatoes as found in the study. These findings are not surprising as several studies have described remarkable differences in food choices between men and women (Maginsh et al. 2022; Yahia et al. 2016; Kamphuis et al. 2015). These could be explained by the reasons for consuming sweetpotatoes in Table 3 where consumption is linked to accessibility, low cost and the fact that all the family likes them. Wanjuu et al. (2019) worked on sweetpotato bread and found that there was a significant relationship between socio‐demographics and overall acceptance of orange‐fleshed sweetpotatoes puree bread.

## 5 Implications for Food Security at the Regional or Global Level

When people have access to the food they need, they become food secure. This food must be of the highest quality to ensure improvement in the nutrition of consumers. The findings point to the fact that, in the near future people who depend on sweetpotatoes for their nutrition may experience food insecurity with the reduction in the production of vitamin A‐rich orange‐fleshed sweetpotatoes. Sweetpotato breeders should therefore consider increasing the taste of the orange‐fleshed sweetpotato variety to enhance the cultivation by farmers. When the nutritious orange‐fleshed sweetpotatoes are made available on the markets, there is a high possibility that households will consume them.

Furthermore, the high level of post‐harvest loss could be reduced by engaging farmers in the production of flour and other products from sweetpotatoes, which would also increase their income levels. However, this requires demographic specifications as there are preferential differences in terms of education, age and marital status.

Lastly, there is a need for education on the nutritional potentials of sweetpotatoes, especially, the orange‐fleshed sweetpotatoes to the younger generation to boost their interest and appetite in consuming sweetpotatoes.

## 6 Conclusion

The study aimed at assessing the effect of gender on the cultivation and consumption of sweetpotato in general and specifically, the orange‐fleshed sweetpotatoes in three districts of the Volta region of Ghana. Generally, except for marketing, activities such as land preparation, fertilizer application, weeding and harvesting are male‐dominated. Though there has been an increase in the cultivation of sweetpotatoes in the three districts, farmers and traders have noted a huge reduction in the cultivation of orange‐fleshed sweetpotatoes from the year 2019 to 2022. The reasons for consuming sweetpotato by individuals and households are affordability, accessibility and likeness. The white‐fleshed sweetpotatoes are preferred over the orange‐fleshed sweetpotatoes due to taste, although consumers are aware that the orange‐fleshed sweetpotatoes contain many nutrients. Processing orange‐fleshed sweetpotatoes into flour or frozen chips (the most preferred forms) will enhance their consumption. Gender did not have any association with the form in which sweetpotatoes are processed. However, the educational level of participants, marital status and age had a significant effect on the preference. While breeders need to improve the taste of the orange‐fleshed sweetpotatoes, processors must provide alternatives that will enhance their consumption. Cultivation and consumption intervention strategies such as policy implementation and nutrition education programs are crucial for increasing the consumption of sweetpotatoes, specifically, the orange‐fleshed sweetpotatoes to enhance food security efforts.

## Conflicts of Interest

The authors declare no conflicts of interest.

## Data Availability

The manuscript contains all data generated from the work.
